# Loss of the calcium channel β_4_ subunit impairs parallel fibre volley and Purkinje cell firing in cerebellum of adult ataxic mice

**DOI:** 10.1111/ejn.13241

**Published:** 2016-04-18

**Authors:** Bruno Benedetti, Ariane Benedetti, Bernhard E. Flucher

**Affiliations:** ^1^ Department of Physiology and Medical PhysicsMedical University of InnsbruckSchöpfstraße 41InnsbruckA‐6020Austria

**Keywords:** ataxia, cerebellar cortex, development, electrophysiology, neuron

## Abstract

The auxiliary voltage‐gated calcium channel subunit β_4_ supports targeting of calcium channels to the cell membrane, modulates ionic currents and promotes synaptic release in the central nervous system. β_4_ is abundant in cerebellum and its loss causes ataxia. However, the type of calcium channels and cerebellar functions affected by the loss of β_4_ are currently unknown. We therefore studied the structure and function of Purkinje cells in acute cerebellar slices of the β_4_^−/−^ ataxic (*lethargic*) mouse, finding that loss of β_4_ affected Purkinje cell input, morphology and pacemaker activity. In adult *lethargic* cerebellum evoked postsynaptic currents from parallel fibres were depressed, while paired‐pulse facilitation and spontaneous synaptic currents were unaffected. Because climbing fibre input was spared, the parallel fibre/climbing fibre input ratio was reduced. The dendritic arbor of adult *lethargic* Purkinje cells displayed fewer and shorter dendrites, but a normal spine density. Accordingly, the width of the molecular and granular layers was reduced. These defects recapitulate the impaired cerebellar maturation observed upon Ca_v_2.1 ataxic mutations. However, unlike Ca_v_2.1 mutations, *lethargic* Purkinje cells also displayed a striking decrease in pacemaker firing frequency, without loss of firing regularity. All these deficiencies appear in late development, indicating the importance of β_4_ for the normal differentiation and function of mature Purkinje cells networks. The observed reduction of the parallel fibre input, the altered parallel fibre/climbing fibre ratio and the reduced Purkinje cell output can contribute to the severe motor impairment caused by the loss of the calcium channel β_4_ subunit in *lethargic* mice.

## Introduction

In cerebellum, presynaptic P/Q‐type calcium channels are made up of the pore‐forming Ca_V_2.1 α_1_ subunit and auxiliary subunits, among which β_4_ and α_2_δ‐2 are the most highly represented (Buraei & Yang, 2010; Schlick *et al*., 2010). Consistent with their joint function in cerebellar neurons, mutation of any one of these calcium channel subunits in mice and humans leads to similar forms of ataxia and epilepsy (Dung & Swigart, 1971; Fletcher *et al*., 1996; Burgess *et al*., 1997; Escayg *et al*., 2000; Barclay *et al*., 2001; Brodbeck *et al*., 2002; Khan & Jinnah, 2002; Pietrobon, 2005; Dolphin, 2012; Noebels, 2012). Although it is well established that dysfunctions of Ca_V_2.1 or α_2_δ‐2 affect the morphology, synaptic input and pacemaker activity of cerebellar Purkinje neurons (Zwingman *et al*., 2001; Matsushita *et al*., 2002; Kodama *et al*., 2006; Walter *et al*., 2006; Lonchamp *et al*., 2009), the specific cerebellar functions of the β_4_ subunit and their causal link to the ataxic phenotype in β_4_ loss‐of‐function mutations remain elusive.

Calcium channel β subunits associate with Ca_V_1 and Ca_V_2 α_1_ subunits. They modulate their current properties and are important for proper membrane expression and subcellular targeting of the channels (Buraei & Yang, 2010; Etemad *et al*., 2014; Campiglio & Flucher, 2015). Consequently β_4_ subunits are important for calcium‐induced neurotransmitter release at synaptic terminals of central nervous system (CNS) neurons (Caddick *et al*., 1999; Lin *et al*., 1999; Wittemann *et al*., 2000). Furthermore, new channel‐independent functions of β_4_ in gene transcription regulation have recently emerged, suggesting potential roles of this protein in regulation of neuronal development and physiology (Tadmouri *et al*., 2012; Etemad *et al*., 2014). Previous studies in the ataxic β_4_^−/−^ mouse model (*lethargic*) suggested that the function of β_4_ in CNS neurons might be redundant and that the lack of β_4_ is largely compensated for by increased expression of other β isoforms (McEnery *et al*., 1998). This notion is consistent with the observation that calcium currents in Purkinje cell (PC) somas are unaffected by loss of β_4_ (Burgess & Noebels, 1999a). However, a redundancy of β_4_ is at odds with the prominent motor impairment in *lethargic* mice (Burgess *et al*., 1997; Khan & Jinnah, 2002) and the functional compensation, which occurs at the PC soma (Burgess *et al*., 1999), might not necessarily extend to dendrites, axons and synapses (Wittemann *et al*., 2000; Obermair *et al*., 2010; Etemad *et al*., 2014) .

Here we applied electrophysiological recordings from PCs in acute cerebellar slices of β_4_^−/−^ *lethargic* mice to test the hypothesis that the calcium channel β_4_ subunit is necessary for normal function of cerebellar cortex networks, where physiological defects upon loss of β_4_ may contribute to the ataxic phenotype in *lethargic* mice. We reasoned that, because β_4_ is an essential subunit of P/Q‐type calcium channels, the deficiencies in PC networks in *lethargic* mice will resemble those previously reported in Ca_V_2.1 mutant mice, unless compensated for by redundant β functions. On the other hand, β_4_‐specific defects will expose essential roles of β_4_ in other Ca_V_ isoforms and channel‐independent functions of β_4_ in the development of cerebellar cortex networks.

## Materials and methods

### Animal handling

All animal procedures were performed according to the guidelines of ethical approval by the European Parliament Directive (Council Directive 2010/63EU and Council of 22 September 2010) for the protection of animal used for scientific purposes and were approved by the Austrian Ministry BMWFW, TWG 2012. Animals were kindly provided by the laboratory of Professor V. Flockerzi, Universität Saarland (Germany). For all animal experiments, the 3Rs system was followed (replacement, reduction and refinement). Experiments were carried out on animals of either sex. To ease the access to food and further promote the fitness and comfort of *lethargic* mice, dry food pellets and hydrating gel was placed on the cage floor and provided *ad libitum*. All mice were bred at least for ten generations into 129/SvJ inbreed background. *Wildtype* and *lethargic* (β4^−/−^) mice were obtained by homozygous mating. The numbers of animals used for the experiments were: 16 juvenile [postnatal day (P)07–P20] wildtype, 20 juvenile *lethargic*, 34 adult (P35–P65) wildtype and 20 adult *lethargic* mice.

### Electrophysiology

After killing the mice by cervical dislocation and after decapitation, scalp and skull bones were gently removed with the help of fine scissors and tweezers. After this, the mouse cerebellum was acutely dissected and immediately chilled (~ 0 °C) in artificial cerebrospinal fluid containing (in mm): NaCl, 125; NaHCO_3_, 26; glucose, 10; KCl, 2.5; NaHPO_4_, 1.25; CaCl_2_, 2; MgCl_2_, 1; pH was adjusted to 7.4 with a saturating carbogen mix (95/5% CO_2_/O_2_). Parasagittal cerebellar slices were cut along the vermis, with a vibratome (Leica VT1200S). After dissection, slices were stored in artificial cerebrospinal fluid at 21.5–22.5 °C (room temperature) for up to 5 h.

Recordings were obtained from visually identified PCs from the anterior lobe, mostly within lobules III and IV. Electrophysiological data were acquired with an Axon 900A amplifier and the software pclamp (Axon Instruments, Union City, CA, USA); sampling rate was 20 kHz. Traces were filtered at 1–4 kHz and analysed with Origin, Microsoft Excel and graphpad‐prism. Series resistances (*R*_s_) were typically about 10 MΩ. *R*_s_ compensation was applied at 60–70%. Recordings with *R*_s_ drift > 10% were discarded. The internal pipette solution contained (in mm): K‐gluconate, 132; EGTA/KOH, 1; MgCl_2_, 2; NaCl, 2; Hepes/KOH, 10; Mg‐ATP, 2; GTP, 0.5; pH was adjusted to 7.2 (Lonchamp *et al*., 2009). Pipette resistance was about 2 MΩ for whole‐cell measurements and 3–7 MΩ for cell‐attached recordings. Parallel fibre (PF) volleys were evoked by unipolar stimulation electrode (glass pipette of about 1 mΩ) in the molecular layer beneath the pial surface. Climbing fibre (CF) responses were evoked by similar stimulation in the granular layer. In the representative recordings of evoked synaptic responses, the stimulation artefacts were removed for clarity of representation. The optimal age range for electrophysiological recording was determined after preliminary excitatory postsynaptic current (ePSC) and inhibitory postsynaptic current (iPSC) measurement between P06 and P20, where the largest increase in PSC frequency occurred before P12 (data not shown). ePSC and iPSC on young mice were sampled at room temperature from mice between P12 and P20, with the same distribution between β_4_^+/+^ and β_4_^−/−^ (P14.5 ± 1.0 and P15.0 ± 0.9, respectively). Pacemaker firing was measured at 34–36 °C. Action potential frequency in young mice was determined in cell‐attached experiments at P14–P15. Action potential frequency in adult mice was recorded between postnatal weeks 5 and 8 (P43 ± 1 for β_4_^+/+^ and P44 ± 1 for β_4_^−/−^); cells from the same mice were also used for morphological analysis. In adult populations no correlation existed between ages and firing frequency (*R* = 0.3, *P* = 0.5 for β4^+/+^ and *R* = −0.6, *P* = 0.1 for β4^−/−^). Evoked postsynaptic currents were recorded at P15 and P55–P65 (P60). Drugs were applied at the following concentrations (mm): (±)‐*trans*‐1‐amino‐1,3‐dicarboxycyclopentane (*trans*‐ACPD; Tocris, Bristol, UK) 0.05; SR 95531 hydrobromide (gabazine; Tocris) 0.01; and 6,7 dinitroquinoxaline‐2,3‐dione (DNQX; Tocris) 0.01, dl‐AP‐5 (AP‐5; Tocris) 0.05.

### Morphological analysis

During whole‐cell patch clamp recordings, Purkinje neurons were dialysed with biocytin via patch‐pipette (Benedetti *et al*., 2011). After 10–20 min the pipette was gently retracted, slices were transferred to 4% sucrose/paraformaldehyde in 0.1 m phosphate buffer, and stored at 4 °C for 24–96 h before staining. Then, slices were thoroughly rinsed in phosphate‐buffered saline, incubated for 1 h in 0.2% Triton‐X100 at room temperature, rinsed and again with phosphate buffer. To block endogenous biotin, slices were treated with the Endogenous Biotin‐Blocking Kit (Molecular Probes, Carlsbad, CA, USA). Streptavidin conjugated with Alexa Fluor 568 (Molecular Probes) was added (1:1000 in phosphate‐buffered saline) and incubated overnight at 4 °C with shaking. Finally, slices were thoroughly rinsed in phosphate buffer and mounted with Vectashield (Vector Laboratories, Burlingame, CA, USA). Labelled neurons were imaged using a confocal microscope (SP5; Leica, Wetzlar, Germany) using a 20× air objective and leica las af software. For morphological analysis, vertical projections of *z*‐stacks were processed with the software las af (Leica) and image j. For Scholl analysis, intersections were counted between dendritic branches and concentric circles, starting at 6 μm from the cell soma and at radial increments of 3 μm, up to 250 μm (maximal radial dendrite extension). Dendritic tracking was performed with the ‘Neuron J’ plugin to image j (courtesy of Dr Erik Meijering, Erasmus University Medical Center Rotterdam). Counting of synaptic spines was performed on higher‐magnification scans (63× glycerol objective) from non‐overlapping portions of proximal (< 100 μm from soma) or distal (> 100 μm from soma) dendrites. The number of spines was then normalized for the length of the analysed dendrite.

### Data analysis and statistics

**Figure 1:**
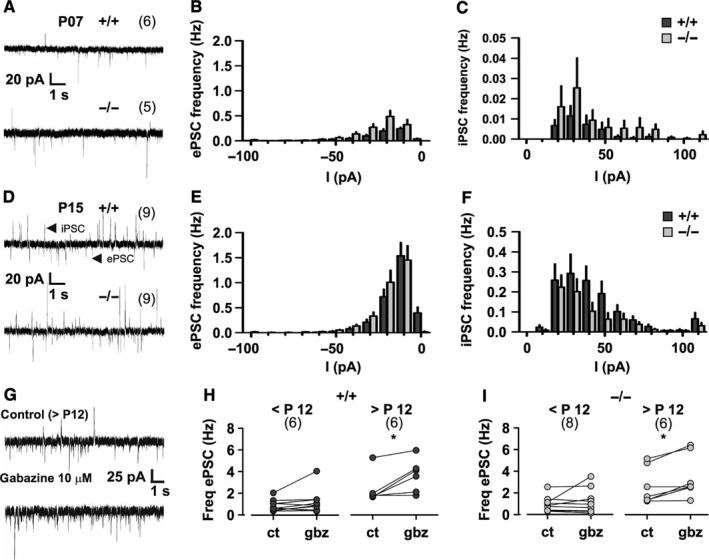
Spontaneous excitatory and inhibitory postsynaptic currents (ePSCs and iPSCs) in young Purkinje neurons from wildtype (+/+) and *lethargic* (−/−) mice. (A, D) Representative recordings from P07 and P15 mice show ePSCs and iPSCs as inward and outward current spikes, respectively. (B, C, E, F) Graphs depicting ePSC and iPSC amplitude and frequency in P07 (B, C) and P15 mice (E, F). (G) iPSCs and ePSCs in control conditions, and in the presence of gabazine; note the absence of iPSCs after gabazine application. (H, I) Mean ePSC frequency in paired recordings before and after gabazine application in +/+ (H) and −/− (I) mice. Note the significant increase in ePSC frequency in both +/+ and −/− mice after P12. Bar graphs indicate mean ± SEM; anova tests were used for comparison of amplitude and frequency as detailed in Table 1. A paired *t*‐test was used in H and I; *P < 0.05. Numbers of independent samples are indicated in parentheses in the figure.

**Figure 2:**
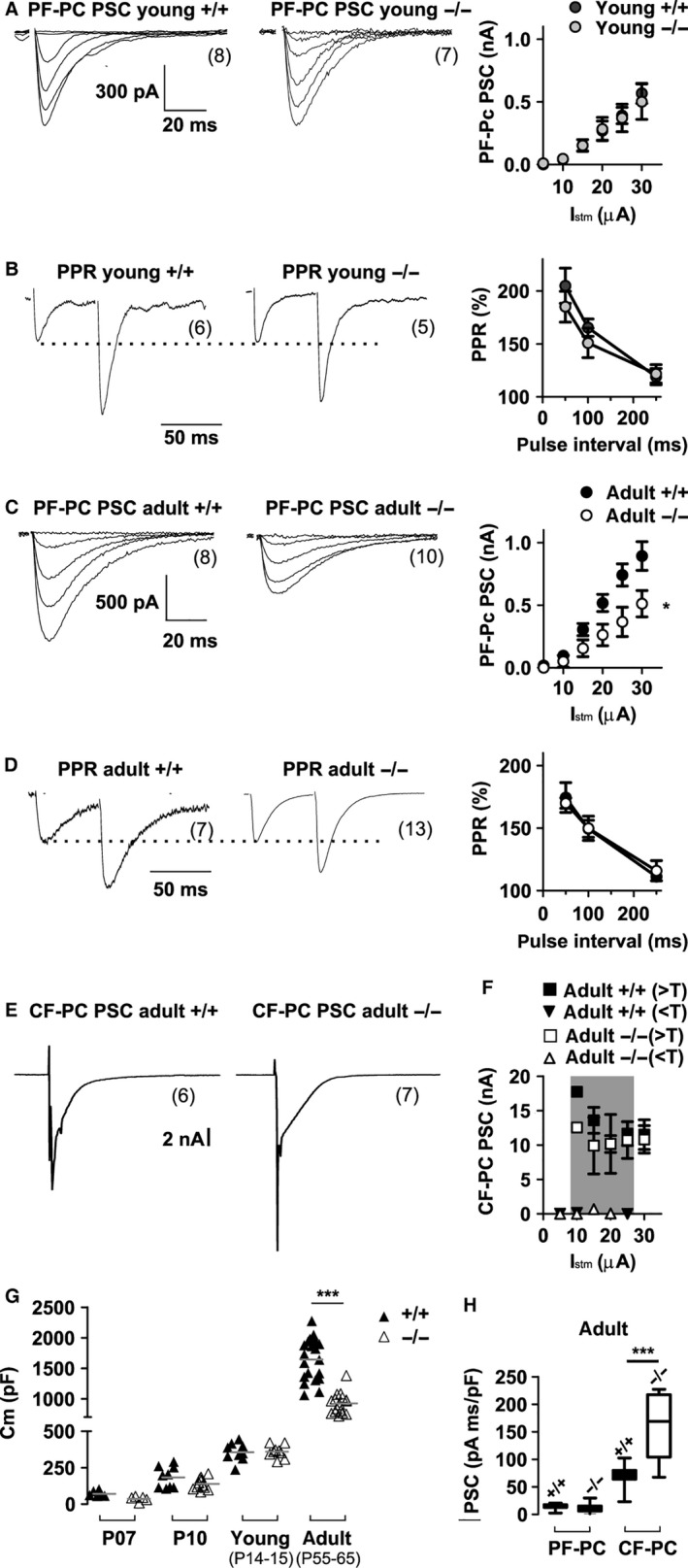
Evoked postsynaptic currents (PSCs) and paired pulse facilitation in cerebellar Purkinje neurons upon stimulation of parallel fibres and climbing fibres in wildtype (+/+) and *lethargic* (−/−) mice. (A–D) Representative PF volley recordings in juvenile (A, B) and adult (C, D) mice; *P < 0.05; two‐way anova. (A, C) PSC amplitude in responses to increasing stimulus intensity displays a significantly higher amplitude in adult +/+ compared to −/− mice (c). (B, D) PPF upon repetitive PF stimulation shows that the PPRs are not different in lethargic mice compared to age‐matched controls (mean ± SEM). (E) Representative PSCs upon CF stimulation. (F) CF–PC PSC all‐or‐none responses display constant amplitude at increasing stimulus intensities above effective threshold (> T, squares) and below threshold (< T, triangles). (G) Differences in whole cell capacitance (Cm) in adult PCs reveals a significantly decreased PC size in −/− mice. (H) Integral of PF–PC and CF–PC PSCs normalized to whole cell capacitance demonstrates that relative to cell size current density of PF–PC is identical in genotypes, but CF–PC current density is significantly increased in −/− mice. Box‐plots show mean, inter‐quartile distribution and 10–90% of the data range. ‘Young’ mice were P14–P15, while ‘adult’ mice were P55–P65. Differences between PF–PC PSCs were measured at any stimulation intensity with two‐way anova test; other differences between multiple populations (G, H) were measured with one‐way anova test followed by Bonferroni post‐test as detailed in Table 2. *P < 0.05; ***P < 0.0001. Numbers of independent samples are indicated in parentheses in the figure.

**Figure 3:**
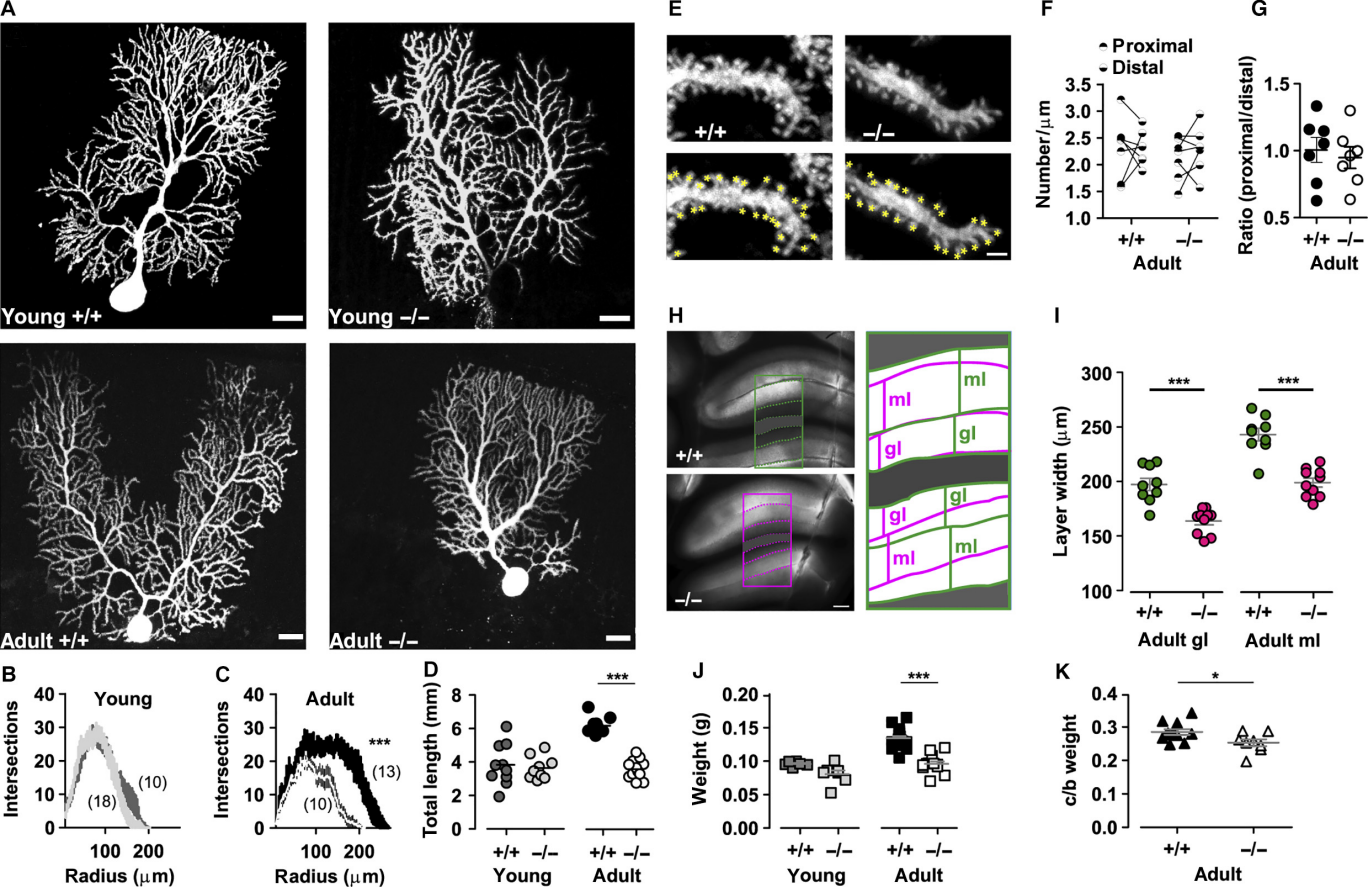
Morphological analysis of Purkinje neurons, granular and molecular layers. (A) Purkinje neurons of juvenile and adult wildtype (+/+) and *lethargic* (−/−) mice, perfused with biocytin during patch‐clamp recording and subsequently fluorescently labelled and analysed with confocal microscopy. Scale bars = 20 μm. (B, C) Graphs of the number of radial intersections of dendrites at increasing distance from the soma (Scholl analysis) in young (B) and adult (C) +/+ and −/− mice. Note the significantly diminished branching of adult −/− PCs. (D) Total dendritic length is significantly increased in dendrites in adult +/+ PCs. (E) High‐magnification images of PC dendrite segments in +/+ and −/− mice with dendritic spines highlighted by yellow marks (lower panels). Scale bar = 1.5 μm. (F) Average spine density in proximal and distal dendritic segments in adult +/+ and −/− neurons; paired measurements in each cell are connected by lines. (G) Ratio between proximal and distal spine density. (H) Brightfield images of acute cerebellar slices from adult +/+ and −/− mice. Scale bar = 250 μm. Masks outlining the granular and molecular layers are expanded and superimposed in the right panel. (I) Average width of adult +/+ and adult −/− granular and molecular layers. Note that both granular and molecular layers are significantly narrower in the cerebellar cortex of −/− mice. (J) Cerebellar weight of young +/+ (dark grey) and young −/− (light grey), adult +/+ (black) and adult −/− (white) mice. (K) Ratio between cerebellum and brain weight (c/b) in adult +/+ (black) and adult −/− (white) mice. Differences in dendritic branching (B, C) were analysed with two‐way anova and numbers of independent samples are indicated in parentheses. Differences between multiple populations were analysed with one‐way anova followed by Bonferroni post‐test (D, F, J, I). A Mann–Whitney test was used in the comparison between adult cerebellum/brain weight ratio and proximal/distal spine ratio (G, K). *P < 0.05, ***P < 0.0001.

Statistical analysis was performed with the software graphpad‐prism 5.03. Statistical significance in the comparison among groups was determined as follows. (i) Normality of data distribution was determined for each group with the Kolmogorov–Smirnoff test. (iia) In case of a normal distribution, a *t*‐test or analysis of variance (anova) + Bonferroni test were used for comparison between, respectively, two or multiple groups. (iib) In case of a non‐normal distribution, Mann–Whitney test or Kruskal–Wallis + Dunn's test were used for comparison between, respectively, two or multiple groups (see Figs 5 and 6). (iii). Two‐way anova was used to compare differences in PF–PC PSC in the presence or absence of β_4_ at each stimulation intensity (see Fig.  2). The type of test employed to analyse each set of data is specified in the figures and tables. Bar graphs represent mean ± standard error of the mean (SEM). Scatter dot plots represent single data points. Plots in which symbols are connected by lines (Figs 1H and I, and 3F) represent paired sets of data. Box plots (Fig. 2) represent mean, interquartile range and 5–95% of range. Numerical *P* values are reported in the tables, where *F* and *P* values are reported for repeated measurements (*F*;* P*). Significance is indicated as: *P < 0.05, **P < 0.001, ***P < 0.0001. The number of independent samples (different cells) is reported in parentheses in the figures and tables. For each type of experiment, measurements were carried out on three or more β4^+/+^ and three or more β4^−/−^ mice, except for the measurements of pacemaker firing in thge presence of DNQX/gabazine (Fig. 5) which were carried out on two animals.

## Results

### Loss of β_4_ does not reduce the spontaneous synaptic input of Purkinje neurons

The β_4_ subunit is known to support Ca_V_2.1 calcium channel activity and thus promotes synaptic transmission in CNS thalamic neurons and in cultured hippocampal neurons (Caddick *et al*., 1999; Wittemann *et al*., 2000). Therefore, loss of β_4_ in *lethargic* (β_4_^−/−^) mice is expected to severely affect the activity of Ca_V_2.1 in cerebellar neurotransmission (Lonchamp *et al*., 2009; Galliano *et al*., 2013). To test this possibility we performed patch clamp recordings of ePSC and iPSC in PCs of acute cerebellar slices. In the whole‐cell configuration the membrane potential of PCs was held at −40 mV in 1‐week‐old mice (P07), or at −70 mV in 2‐week‐old mice (P15). At P07 (Fig. 1A) postsynaptic currents principally comprised ePSCs, while iPSCs were very rare (Fig. 1B and C). At P15 (Fig. 1D) ePSCs and iPSCs occurred at increased frequency (Fig. 1E and F; *P *> 0.04). Importantly, at both ages we found no difference in the frequency of ePSCs or iPSCs between *lethargic* and age‐matched control cerebellar slices (Table 1). Current amplitude was also not impaired by the lack of β_4_; while the ePSC amplitude was slightly increased in *lethargic* mice (Table 1), the iPSC amplitude was not changed.

**Table 1:** Postsynaptic current (PSC) frequency and amplitude

	Frequency (Hz)		*n*	Amplitude (pA)		*n*	Postnatal days
	ePSC	iPSC		ePSC	iPSC		
^+/+^P07	0.8 ± 0.2	0.15 ± 0.07	6; 6	25 ± 1	29 ± 4	6; 4	7.0 ± 0.0
^−/−^P07	0.6 ± 0.1	0.13 ± 0.07	5; 5	35 ± 2	54 ± 17	5; 3	6.8 ± 0.2
*P* *	0.4	0.9		0.009	0.15		
anova:* F*;* P*	–	–		19.73; < 0.0001	2.1; 0.1		
Bonferroni	–	–		< 0.001	–		
^+/+^P15	3.0 ± 0.6	1.3 ± 0.4	9; 9	18 ± 1	49 ± 6	9; 9	15.0 ± 0.8
^−/−^P15	3.2 ± 0.5	0.8 ± 0.2	9; 9	25 ± 1	35 ± 5	9; 9	16.0 ± 0.9
*P* *	1.0	0.4		0.0005	0.1		
anova:* F*;* P*	–	–		19.73; < 0.0001	2.1; 0.1		
Bonferroni	–	–		< 0.05	–		

Mean ± SEM are reported. *P values refer to unpaired *t*‐test. anova (*F*;* P*) values refer to multiple comparisons between groups of different age, and Bonferroni post‐test (*P*) amongst the same data. –, not tested; *n*, number of samples.

Because excitatory input from granule cells to PCs is subject to tonic GABAergic inhibition (Hamann *et al*., 2002), we wondered whether this inhibitory activity could have masked differences in excitatory synaptic activity. Therefore, in a subset of experiments ePSCs were recorded in the presence of the selective GABA receptor blocker gabazine (Fig. 1G). In cerebellar slices from animals older than 12 days, gabazine (10 μm) completely blocked the iPSCs and concomitantly led to a significant increase of ePSC frequency (paired *t*‐test; *P* = 0.03, β_4_^+/+^; *P* = 0.01, β_4_^−/−^). Younger cerebellar slices (< P12) were not affected (*P* = 0.2, β_4_^+/+^; *P* = 0.4, β_4_^−/−^). Notably, the effect of gabazine was not different between wildtype (Fig. 1H) and *lethargic* neurons (Fig. 1I). Together these data demonstrate that loss of β_4_ does not impair the spontaneous synaptic transmission onto PCs. Considering the importance of P/Q‐type calcium channels for presynaptic function, this suggests that the lack of β_4_ in cerebellar synapses of *lethargic* mice may be compensated for by other β isoforms, as previously suggested (McEnery *et al*., 1998; Burgess *et al*., 1999).

### Loss of β_4_ in adult lethargic mice specifically affects PF volley but not paired‐pulse facilitation (PPF)

Although β_4_ loss did not impair spontaneous synaptic activity, it might affect PF input via postsynaptic mechanisms, similar to what was previously observed in Ca_v_2.1 mutations (Matsushita *et al*., 2002; Kodama *et al*., 2006). We therefore measured postsynaptic currents evoked by synchronous synaptic release from PFs (PF–PC PSCs). Electrical pulses increasing from 5 to 30 μA were locally delivered in the molecular layer. In both young and adult PCs these stimuli triggered PF–PC PSCs of increasing amplitudes (Fig. 2A and C; Table 2). In cerebellum of young *lethargic* mice the amplitudes of the evoked PF–PC PSCs were comparable to those recorded in wildtype controls. As PPF is a measure of presynaptic calcium signalling and regulation of release probability, we also recorded PSCs in response to two consecutive stimuli of equal strength (Fig. 2B). In young wildtype and *lethargic* mice the paired‐pulse ratios (PPRs) of PF–PC synapses were not significantly different. Thus, in young mice the function of PF–PC synapses and total synaptic input from PFs is apparently not affected by the lack of the calcium channel β_4_ subunit.

**Table 2:** PF–PC PSC amplitude, paired pulse ratios and CF–PC PSC amplitude; see also Fig. 2

	PF–PC PSC (STM = 30 μA)			PF–PC PPR	CF‐Pc PSC	
	nA	nA/pF	ʃPSC/Cm (pA.ms)/pF		nA	ʃPSC/Cm (pA.ms)/pF
^+/+^ young (P14–15)	0.6 ± 0.1 (*n* = 8)			209 ± 21 (*n* = 7)		
^−/−^ young (P14–15)	0.6 ± 0.1 (*n* = 6)			185 ± 14 (*n* = 5)		
*P* *	0.7			0.7		
^+/+^ adult (P55–65)	0.9 ± 0.1 (*n* = 8)	0.54 ± 0.05 (*n* = 8)	15 ± 2 (*n* = 8)	182 ± 10 (*n* = 7)	11.5 ± 2.2 (*n* = 6)	69 ± 9 (*n* = 6)
^−/−^ adult (P55‐65)	0.5 ± 0.1 (*n* = 10)	0.61 ± 0.15 (*n* = 10)	12 ± 3 (*n* = 10)	170 ± 4 (*n* = 13)	10.8 ± 2.0 (*n* = 7)	158 ± 24 (*n* = 7)
*P* *	0.03	0.7	0.5	0.4	0.9	0.009
anova:* F*;* P*	–	–	34; < 0.0001	–	–	34; < 0.0001
Bonferroni	–	–	ns	–	–	< 0.0001
A‐2 (S): *F*;* P* A‐2 (T): *F*;* P*	5.3; 0.03 58; < 0.001	–	–	–	–	–

Mean ± SEM are reported. *P values refer to unpaired *t*‐test. anova (*F*;* P*) values refer to multiple comparison between all evoked responses (PF–PC PSCs and CF–PC PSCs) normalized for cell capacitance in adult mice followed by Bonferroni post‐test. ns, not significant; –, not tested. Two‐way anova (*F*;* P*) analyses the differences between PF–PC PSC amplitudes in the presence or absence of β_4_ (A2‐S) at each stimulation intensity (A2‐T; intensity = 5–30 μA).

In contrast, evoked PF–PC PSCs were significantly decreased in adult *lethargic* cerebellum (Fig. 2C). Under the assumption that axonal excitability, axonal density and probability of PF–PC connection are not altered, changes in the PF–PC PSC in *lethargic* mice would imply altered synaptic transmission. Here PSC amplitudes evoked by 30‐μA pulses were about half the size of PSCs in age‐matched wildtype controls (−/−, 0.5 ± 0.1 nA; +/+, 0.9 ± 0.1 nA; *P* = 0.03). Nevertheless, PPRs in adult *lethargic* mice were still unaltered (Fig. 2D; *P* = 0.4). Thus, the loss of β_4_ dramatically decreased the total synaptic input from PFs in adult but not in juvenile *lethargic* mice. However, the unaltered release probability indicates that this dysfunction does not arise from an impeded presynaptic function in PF–PC synapses.

Because these results suggested a postsynaptic mechanism affecting excitatory neurotransmission in PCs of adult *lethargic* mice, we next examined synaptic input from CFs. Electrical stimuli in the granular layer typically evoked large all‐or‐none postsynaptic currents with effective stimulation thresholds between 10 and 25 μA (Fig. 2E and F). In the cerebellum of wildtype and *lethargic* mice, the amplitudes of CF–PC PSCs were not significantly different from each other (Fig. 2F; Table 2, *P* = 0.9), equal to 10.5 ± 2.1 and 10.8 ± 2.0 nA, respectively, at a stimulus intensity of 30 μA. This indicates that, contrary to PF input, β_4_ is not critically involved in shaping the synaptic input of CFs to PCs.

In our whole‐cell recordings, we noticed that the cell capacitance of adult β_4_^−/−^ PCs was significantly smaller than that of adult β_4_^+/+^ neurons (−/−, 925 ± 42 pF; +/+, 1643 ± 70 pF; *P* < 0.0001; Fig. 2G). No difference was detected at P15 (*P* = 0.8) and in younger mice (P07, P10). When PF–PC PSCs were normalized to the capacitance of each respective neuron, the resulting current densities were not significantly different (*P* = 0.7) between *lethargic* and wildtype mice. Similarly, the integrals of PF–PC PSC/whole cell capacitance of both genotypes were not significantly different (*P* = 0.5; Fig. 2H; Table 2). Thus, in *lethargic* mice the reduction of the PF–PC input matches the decrease in PC size. Integrals of CF–PC PSCs instead did not correspond to the decreased PC cell capacitance. Consequently, the density of CF–PC PSCs was significantly increased in the smaller *lethargic* PCs compared to wildtype controls (Fig. 2H; *P* < 0.01), and the resulting CF–PC PSC/PF–PC PSC ratio was smaller in *lethargic* mice. Furthermore, this decrease in PC capacitance and the associated decrease of PF–PC input in adult *lethargic* mice suggests that the loss of the calcium channel β_4_ subunit affects the late differentiation of the dendritic compartment of PCs.

### Loss of β_4_ affects PC dendritic morphology in adult mice

To further examine the differentiation of PCs dendritic compartment, we analysed the dendritic shape and length in wildtype and *lethargic* PCs. The neurons were dialysed with biocytin via the patch pipette during whole‐cell recordings in acute cerebellar slices. Streptavidin staining of the labelled cells revealed the structure of the complete dendritic arbor (Fig. 3A). Using Scholl analysis we quantified dendritic branching and extension. The overall appearance, size and branching of juvenile *lethargic* PC dendrites was comparable to that of controls (Fig. 3A and B; *F* = 0.4, *P* = 0.5, *n*: +/+ = 18, *n*: −/− = 11). By contrast, in adult *lethargic* mice dendritic branches were fewer and shorter than in age‐matched wildtype controls (Fig. 3B and C; *F* = 22.3, *P* = 0.0001 *n*: +/+ = 10, *n*: −/− = 11). However, weeping‐willow‐like structures (Zwingman *et al*., 2001), or missing portions of the dendritic arbor (Kodama *et al*., 2006) were not observed. Dendrites were also counted within three concentric annular areas centred at the soma (proximal = 0–70 μm; medial = 70–120 μm; distal = 120–200 μm). The greatest differences occurred in the distal (*P* < 0.0001) and medial circle (*P* = 0.01). In the proximal circle, the number of dendrites was comparable in cerebella of *lethargic* and wildtype mice (*P* = 0.1). Total dendrite lengths were equal in young *lethargic* and wildtype mice (−/−, 3.6 ± 0.2 mm; +/+, 3.8 ± 0.4 mm; *P* = 0.7; Fig. 3D). In adults, however, *lethargic* dendrites were significantly shorter than in control PCs (−/−, 3.1 ± 0.1 mm; +/+, 5.4 ± 0.1 mm; *P* < 0.0001; unpaired *t*‐test). In agreement with the electrophysiological data described above, these results reveal a severely retarded differentiation of distal PC dendrites in the absence of β_4_.

If this deficiency in the PC dendrites in *lethargic* cerebellum arises from a diminished number of PFs and sparser synaptic contacts, a decrease in spine density would also be expected (Oda *et al*., 2011). Therefore, synaptic spines were counted in non‐overlapping dendritic segments of wildtype and *lethargic* PCs in proximal and distal dendrites (Fig. 3E). Spine density in β_4_^+/+^ and β_4_^−/−^ neurons was comparable in proximal and distal dendrites (proximal: +/+, 2.3 ± 0.2/μm; −/−, to 2.1/μm; *P* = 0.6; distal: +/+, 2.3 ± 0.1/μm; −/−, 2.3 ± 0.2/μm; *P* = 0.8. Fig. 3F). Also the ratios of spine density in proximal to distal spines were not significantly different in the two genotypes (+/+, 1.01 ± 0.09; −/−, 0.95 ± 0.08; *P* = 0.6. Fig. 3G). Evidently, although the size of the distal dendritic arbor is substantially decreased in *lethargic* PCs, the density of synaptic spines on the remaining dendritic branches is unchanged.

The reduced size of the dendritic arbor is expected to result in a reduced width of the molecular layer. Furthermore, a decreased synaptic input from PFs may originate from a smaller number of granule cells. To examine these possibilities, we measured the widths of the molecular and granular cell layers in wildtype and *lethargic* acute cerebellar slices (Fig. 3H). Indeed, the widths of both the molecular and the granular cell layers were significantly reduced in lethargic mice (molecular layer: +/+, 240 ± 6 μm; −/−, 200 ± 6 μm; granular layer: +/+, 200 ± 4 μm; −/−, 160 ± 4 μm; *P* < 0.0001; Fig. 3I). Thus, the lack of β_4_ not only affects the size of the dendritic arbor of PCs and the thickness of the molecular layer, it probably also causes a reduction in the number of granule cells. This probably results in reduced PF input to PCs. Changes in the thickness of the molecular and granular cell layers were accompanied by a significant weight reduction of the cerebellum (*P* < 0.0001) and by a significant decrease in the proportion of cerebellar to whole‐brain weights (*P* = 0.029) of adult *lethargic* mice compared to wildtype (Fig. 3K and J), indicative of widespread effects of β_4_ loss on brain development.

### Lack of the calcium channel β_4_ subunit impairs the development of high PC pacemaker frequency but not of firing regularity

**Figure 4:**
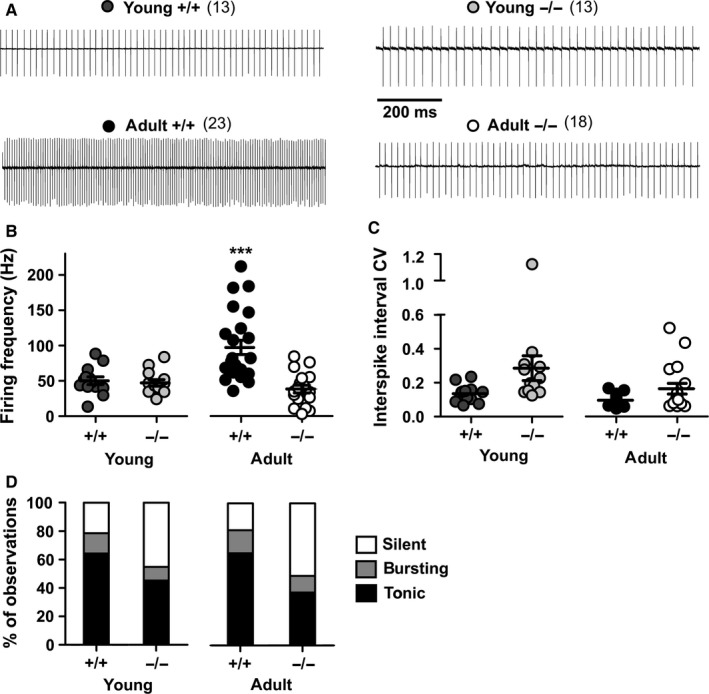
Pacemaker firing of Purkinje neurons in young and adult wildtype (+/+) and *lethargic* (−/−) mice. (A) Representative cell‐attached recordings; vertical deflection of baseline represents individual action potentials. (B, C) Average pacemaker firing frequency (B) and coefficient of variation of the inter‐spike interval (C) in young and adult +/+ and −/− PCs. Each mark in the scatter plots represents individual average firing properties. Bars indicate mean ± SEM. Note that the frequency of adult wildtype mice is significantly higher than that of *lethargic* mice. (D) Bar graph indicating the probability and the proportion between independent observations of silent, bursting and tonic firing cells in young and adult +/+ and −/− mice. Differences between firing frequencies were analysed with one‐way anova followed by Bonferroni post‐test and differences between CV (not normally distributed) were analysed with Kruskal–Wallis test, followed by Dunn's post‐test. ***P < 0.0001. Numbers of independent samples are indicated in parentheses in the figure.

Finding that the lack of β_4_ in *lethargic* mice specifically reduces the PF input and dendritic morphology of cerebellar PCs, we reasoned that this might impair the output of these neurons. Therefore, spontaneous PC pacemaker activity was recorded in acute cerebellar slices from young and adult mice. At P15, locomotion is acquired and the cerebellar network is not fully developed (Altman, 1972). At 1 month of age, locomotion and the cerebellar networks are mature. Figure 4A shows tonic action potential firing of young and adult PCs from cell‐attached recordings in acute cerebellar slices of wildtype and *lethargic* mice. Firing occurred at relatively low frequencies in young mice (P14–15) of both genotypes (+/+, 50 ± 5 Hz; −/−, to 47 ± 5 Hz; *P* = 0.7; Fig. 4B). Firing frequencies increased to 97 ± 10 Hz in wildtype adults (P35–60). However, in adult *lethargic* mice PC firing frequencies failed to increase (38 ± 5 Hz; Fig. 4A and B) and were equal to values measured in the young animals. Accordingly, the age‐related increase in firing frequency was highly significant only in the wildtype mouse cerebellum (*P* = 0.002), but not in the *lethargic* mouse cerebellum (*P* = 0.2). Furthermore, the firing frequency of wildtype adults was significantly higher than that of *lethargic* adults (*P* < 0.0001). Thus, in the absence of β_4_, functional maturation of the PC pacemaker firing frequency fails after P15. As Purkinje axons are the only cortical efferent, low PC firing rates correspond to decreased cerebellar cortical output.

To also quantify the regularity of PC action potential firing in *lethargic* and wildtype mice, we calculated the coefficient of variation (CV) of the inter‐spike intervals (Fig. 4C). In young neurons CV was increased from 0.13 ± 0.01 in β_4_^+/+^ to 0.28 ± 0.07 in β_4_^−/−^ neurons (*P* = 0.007, unpaired *t*‐test). However, after a Bonferroni post‐test, the difference between CV of young mice or between CV of adult mice was no longer significant (Table 3). In adults, the firing regularity had improved in both wildtype and *lethargic* mouse neurons (+/+, *P* = 0.02; −/−, *P* = 0.008; unpaired *t*‐test); the difference in CV between the two genotypes was not statistically significant (0.09 ± 0.01 and 0.16 ± 0.03, respectively; *P* = 0.2). Together these analyses demonstrate that in adult *lethargic* cerebellum the firing rate of PCs is substantially reduced without a concomitant loss of firing regularity. In wildtype and *lethargic* mice we observed tonic firing, bursting and silent PCs. In young and in adult wildtype mice, the tonic firing PCs accounted for 65% and silent PCs for 20% of the whole cell population. In *lethargic* mice, tonic firing was instead limited to 45% of the young and 37% of the adult PC cell population. This was coincident with an increased number of silent PCs to 45 and 50% in young and adult *lethargic* mice, respectively. In conclusion, not only were the tonic firing rates were reduced but also the number of active neurons was diminished by the loss of β_4_, which could contribute to depression of cortical output. The burst firing mode occurred rarely and the proportion of bursting neurons in wildtype and *lethargic* mice (10–15% of the total cell population) was virtually the same at any age.

**Table 3:** Pacemaker firing frequency and coefficient of variation

	Frequency (Hz)	CV	*n*
^+/+^ young (P14–15)	50 ± 5	0.135 ± 0.014	13
^−/−^ young (P14–15)	47 ± 5	0.285 ± 0.073	13
*P*	0.7*	0.007†	
^+/+^ adult (P35–60)	97 ± 10	0.096 ± 0.006	23
^−/−^ adult (P35–60)	38 ± 5	0.164 ± 0.031	18
*P*	< 0.0001*	0.2†	
*P* young vs. adult ^+/+^	0.002*	0.02†	
*P* young vs. adult ^−/−^	0.2*	0.008†	
Multiple comparison (one‐way anova = * or K‐W = †) *F*;* P*	13.05*; < 0.0001	21.6 < 0.0001†	
*Post‐hoc* (young ^+/+^ vs. ^−/−^)	ns*	ns†	
*Post‐hoc* (adult ^+/+^ vs. ^−/−^)	< 0.0001*	ns†	
*Post‐hoc* (young ^+/+^ vs. adult ^+/+^)	< 0.01*	ns†	
*Post‐hoc* test (young ^−/−^ vs. adult ^−/−^)	ns*	*P* < 0.5†	

Mean ± SEM are reported. *P* (*) values refer to unpaired *t*‐test. Multiple comparison refers to one‐way anova (*P*;* F*) and Bonferroni post‐test. The coefficient of variation (CV) of −/− populations was not normally distributed, and therefore significance was tested with Mann–Whitney test and Kruskal–Wallis test (K‐W statistic; *P*) followed by Dunn's multiple comparison test (^†^). ns, not significant; –, not tested.

### High pacemaker firing rates are not dependent on tonic glutamatergic neurotransmission

**Figure 5:**
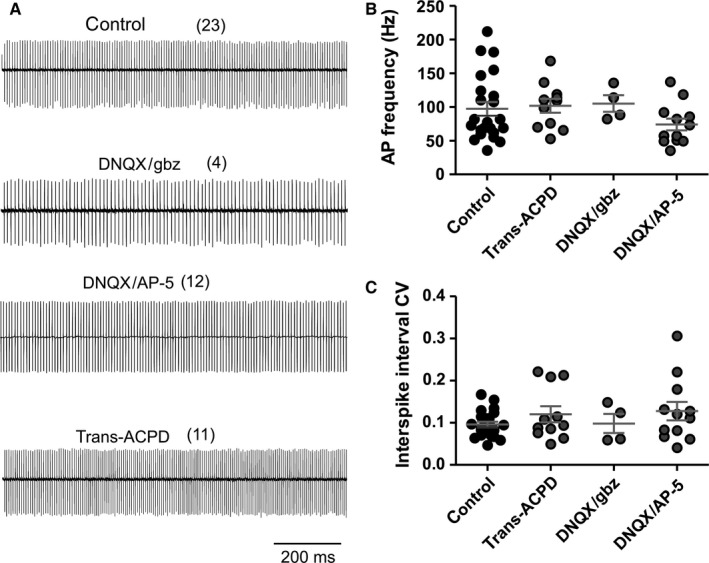
Pacemaker firing of wildtype adult Purkinje neurons in control conditions and after blockage of fast synaptic neurotransmission. (A) Representative cell‐attached recording from PCs in untreated control slices, and in the presence of: 10 μm 6,7 dinitroquinoxaline‐2,3‐dione and 10 μm SR 95531 (DNQX/gbz); 10 μm DNQX and 50 μm AP‐5 (DNQX/AP‐5); or 50 μm *trans*‐ACPD. (B, C) Average pacemaker firing frequency (B) and coefficient of variation of the inter‐spike interval (C) in untreated neurons and after pharmacological treatment. Differences between action potential firing in the presence of drugs and untreated controls were analysed with unpaired *t*‐test for normally distributed samples (frequencies) or Mann–Whitney test for non‐normally distributed samples (CV). Numbers of independent samples are indicated in parentheses in the figure.

To examine whether the decreased PC tonic firing rates might be related to the observed reduction of PF–PC, we recorded spontaneous firing of PCs in cerebellar slices of wildtype and *lethargic* mice upon block of tonic neurotransmission (Fig. 5A). We used bath application of DNQX and gabazine, DNQX and AP‐5, or DNQX and *trans*‐ACPD for 15–60 min to block α‐amino‐3‐hydroxy‐5‐methyl‐4‐isoxazolepropionic acid (AMPA), *N*‐methyl‐d‐aspartate (NMDA), gamma‐aminobutyric acid (GABA)_A_ and metabotropic glutamate receptors. However, none of these conditions affected the firing rates or firing regularity of PCs in wildtype (Fig. 5B, Table 4) or *lethargic* cerebellum. Thus, altered synaptic input is probably not involved in the short‐ to medium‐term modulation of PC pacemaker firing frequency and therefore unlikely to be the cause for the differences in firing frequency observed in adult wildtype and *lethargic* mice. However, these experiments do not exclude possible long‐term effects of altered neurotransmission in *lethargic* mice and a critical involvement of β_4_ in other potential modulatory pathways.

**Table 4:** Pacemaker firing frequency and coefficient of variation after pharmacological blockage of glutamate and/or GABA receptors; see also Fig. 5

	Frequency (Hz)	CV	*P*	*n*
^+/+^DNQX/gbz	105 ± 12	0.098 ± 0.022	0.3; 0.9	4
^+/+^DNQX/AP‐5	74 ± 9	0.128 ± 0.021	0.15; 0.4	12
^+/+^ *trans*‐ACPD	102 ± 10	0.120 ± 0.019	0.2; 0.5	11

Average ± SEM are reported. *P* values refer to unpaired *t*‐test. These experiments were carried out in adult mice (P35–60).

### Afterhyperpolarization is not affected by the loss of β_4_

**Figure 6:**
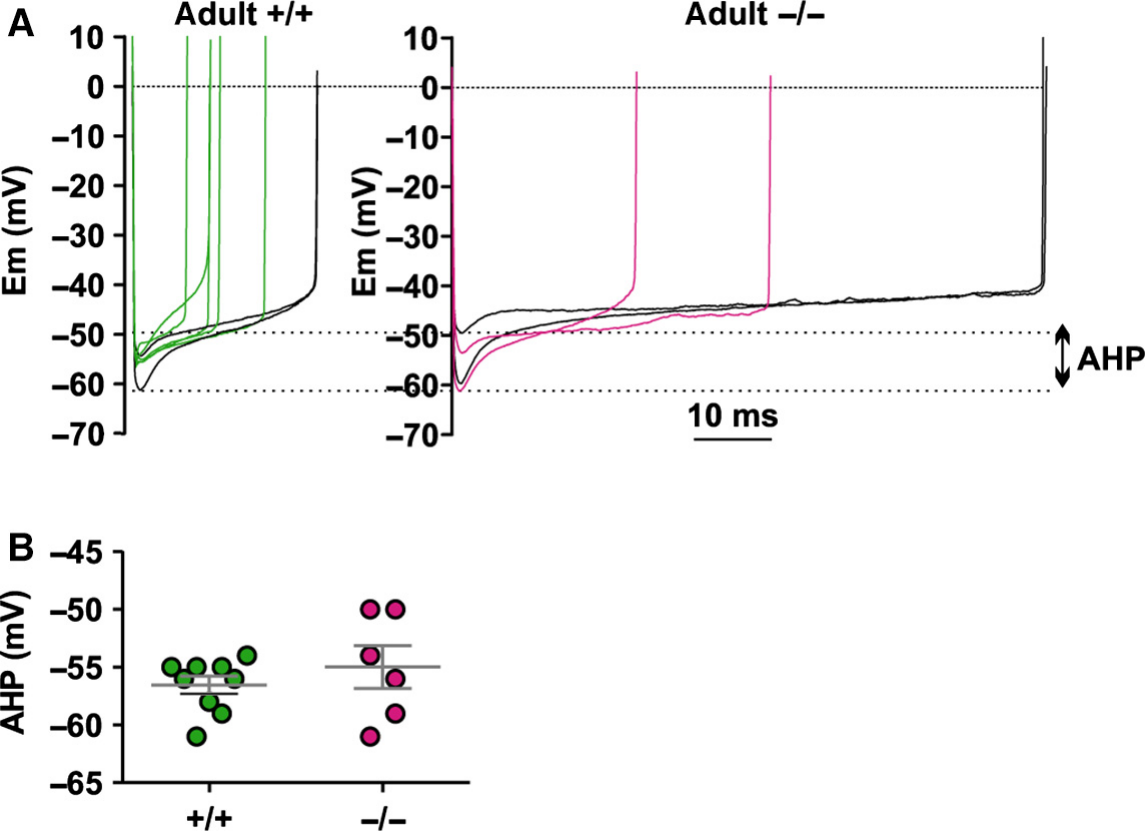
Whole‐cell current clamp recordings of afterhyperpolarizations (AHPs) in adult PCs. (A) Representative action potential inter‐spikes from regularly firing cells in adult wildtype (+/+) and *lethargic* (−/−) mice; dotted lines indicate the range of AHP. Note that action potentials with the same inter‐spike duration produce extremely different amplitudes of AHP (highlighted in black). (B) Scatter plot of average AHP in +/+ and −/− pacemaker firing neurons. Each mark represents an individual average AHP. Differences between AHP were analysed with unpaired *t*‐test.

Reduction of PC pacemaker rates occurs in an ataxia mutation of the BK calcium‐activated potassium channel (Sausbier *et al*., 2004). This is associated with a prominent decrease in potassium currents during afterhyperpolarization (AHP). If sparser pacemaker firing in *lethargic* PCs involved BK potassium currents, this would be revealed by smaller AHP amplitudes. However, whole cell recordings from regularly firing wildtype or *lethargic* neurons showed normal AHP with negative peaks of −56.6 ± 0.7 and −55.0 ± 1.9 mV, respectively (Fig. 6; *P* = 0.4). Furthermore, longer inter‐spike intervals among our samples were not necessarily associated with smaller AHP. Therefore, small AHP derived from loss of BK potassium currents does not contribute to the *lethargic* phenotype.

## Discussion

**Figure 7:**
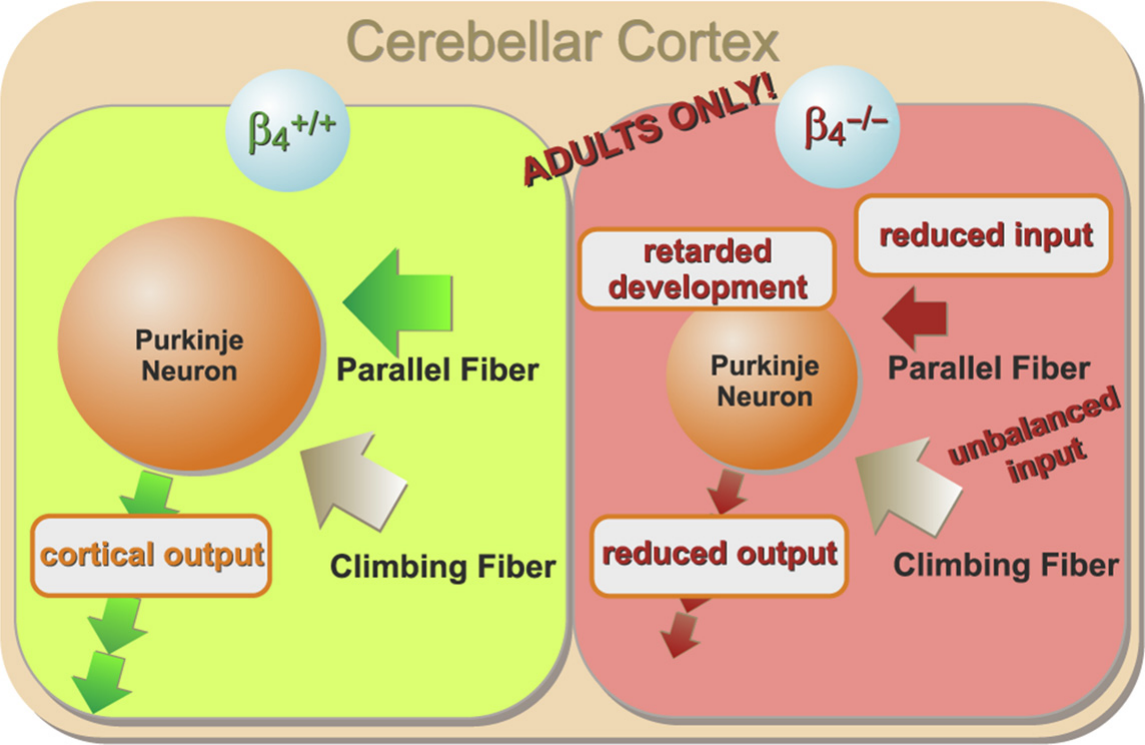
Changes in cerebellar cortical network caused by the lack of β_4_ in adult *lethargic* mice. In the absence of β_4_ the input from PFs is reduced, whereas the input from CFs is spared. Thus, the PC inputs are unbalanced. The size of PCs (dendritic branching) and the width of the granular cell layer are reduced. The firing frequency of PCs and thus cerebellar output is reduced. All these deficits affect only adult and not juvenile *lethargic* mice, consistent with a developmental retardation of cerebellar cortical networks in *lethargic* mice.

Loss of the calcium channel β_4_ subunit causes motor impairment (Khan & Jinnah, 2002) with striking similarities between the *lethargic* (β_4_^−/−^) mouse and mouse models with Ca_v_2.1‐related forms of ataxia (Fletcher *et al*., 1996; Burgess & Noebels, 1999a,b; Barclay *et al*., 2001; Guida *et al*., 2001; Pietrobon, 2002). However, past investigations of physiological and pathophysiological roles of β_4_ in the brain yielded conflicting results. Depending on the brain region or experimental system used β_4_ was found either to be essential for normal neuronal function (Caddick *et al*., 1999; Lin *et al*., 1999; Wittemann *et al*., 2000), potentially relevant (McEnery *et al*., 1998), or even entirely redundant with other β isoforms (Burgess *et al*., 1999). Apparently the context of the brain region and the specific neuronal network is critical for understanding the role of the β_4_ subunit in the etiology of the *lethargic* phenotype (Caddick *et al*., 1999). Because of the importance of the cerebellar cortex in motor control we examined here (i) whether the loss of β_4_ recapitulates the effects of loss of Ca_v_2.1 on cerebellar function and morphology, (ii) which functions of PC networks are primarily impaired by the loss of β_4_ and (iii) how this may contribute to the ataxic phenotype in *lethargic* mice. The present slice electrophysiology study demonstrates that the lack of the calcium channel β_4_ subunit causes a decreased late development in cerebellar cortical networks, characterized by reduced PF–PC input, shorter PC dendrites and depressed PC output (Fig. 7). This is the first study addressing the physiological role of β_4_ and its involvement in the etiology of ataxia in the context of native PC networks in acute cerebellar slices.

### Does loss of the β_4_ subunit affect known functions of Ca_v_2.1 in the cerebellum?

Because the β_4_ subunit is the primary partner of Ca_V_2.1 in cerebellum and loss‐of‐function mutations of both calcium channel subunits result in ataxia (Burgess *et al*., 1997; Burgess & Noebels, 1999a; Escayg *et al*., 2000; Guida *et al*., 2001; Pietrobon, 2002; Buraei & Yang, 2010; Schlick *et al*., 2010), it is reasonable to expect that loss of either one of these proteins affects the same brain functions. Indeed, the loss of β_4_ in the *lethargic* mouse recapitulates a number of impairments of cerebellar function and morphology previously observed in the ataxic Ca_v_2.1 mouse models *rocker*,* tottering* and *purky* (Zwingman *et al*., 2001; Matsushita *et al*., 2002; Kodama *et al*., 2006; Erickson *et al*., 2007; Mark *et al*., 2011). These common impairments encompass a retarded cerebellar development, smaller PC dendrites, PF depression, the resulting imbalance between PF and CF input to PCs, and the late onset of the disease phenotype. Moreover, both the *lethargic* and the *rocker* mice show a normal synaptic density in PC dendrites (Kodama *et al*., 2006), although this was not observed in several other ataxic models (Kashiwabuchi *et al*., 1995; Kurihara *et al*., 1997; Oda *et al*., 2011). This similarity indicates that altered function of cerebellar P/Q‐type calcium channels may contribute to the *lethargic* phenotype. Importantly, it further demonstrates that β_4_ is not functionally redundant, as its loss cannot be fully compensated for by other β isoforms.

On the other hand, other cerebellar functions are differently affected by the loss of Ca_v_2.1 and β_4_. For example general presynaptic loss of Ca_v_2.1 affects the spontaneous synaptic activity (Lonchamp *et al*., 2009; Galliano *et al*., 2013) and alters release probability (Maejima *et al*., 2013). However, in the *lethargic* mouse cerebellum we found that spontaneous synaptic activity and PPF of PF–PC PSCs were not reduced. In theory, the lack of a presynaptic phenotype might indicate compensation for the lost β_4_ function in PF–PC synapses. However, only complete knockout of Ca_v_2.1 in cerebellar granule cells affected presynaptic PF–PC function (Lonchamp *et al*., 2009; Galliano *et al*., 2013; Maejima *et al*., 2013), whereas partial loss of Ca_v_2.1 function did not show a presynaptic phenotype (Matsushita *et al*., 2002; Kodama *et al*., 2006). Apparently the safety margin with regard to calcium influx in this synapse is very high (Matsushita *et al*., 2002), so that a possibly small reduction of P/Q‐type channels caused by the lack of β_4_ may have no functional consequence. By contrast, the spontaneous ePSC amplitude in juvenile *lethargic* mice was increased. While this together with unaltered PPR does not suggest impairment of presynaptic P/Q‐type channels, the slight increase in the *lethargic* PSC amplitude may relate to more subtle changes in the formation of presynaptic channel complexes (Iwasaki *et al*., 2000) upon loss of β_4_. The situation in the cerebellum is probably different from that in other brain regions as the loss of β_4_ resulted in reduced presynaptic function in cultured hippocampal neurons (Wittemann *et al*., 2000; Xie *et al*., 2007), as well as in whole‐brain synaptosome preparations (Lin *et al*., 1999). Moreover, even though the PF PSC was decreased in the absence of β_4_, the unaltered PF PPR and the presence of all‐or‐none CF responses suggest that unlike Ca_v_2.1^−/−^, β_4_^−/−^ has little relevance for heterosynaptic competition between PFs and CFs or for homosynaptic competition among CFs (Miyazaki *et al*., 2004).

Notably, the effects of the loss of β_4_ in *lethargic* mice were not limited to deficiencies of cerebellar functions previously observed in Ca_v_2.1 mutants. We also observed a dramatically decreased PC pacemaker frequency, while the regularity of pacemaker firing was maintained. A similar deficiency of PC pacemaker activity was not observed upon Ca_v_2.1 mutation (*leaner*) nor in closely related (*ducky*) forms of ataxia where both controls and mutants had average firing rates between 75 and 115 Hz (Sausbier *et al*., 2004; Walter *et al*., 2006). In contrast, lethargic PCs had firing rates of about 40 Hz, which is significantly reduced compared to wildtype controls which fired at about 100 Hz. In *lethargic* mice, the β_4_‐specific dysfunction of PC firing suggests a critical function of this protein in the context of another type of calcium channel that cannot be compensated for by alternative β isoforms (McEnery *et al*., 1998; Burgess *et al*., 1999). Alternatively, reduced cerebellar PC firing rates as well as lower number of constitutively active neurons could result from a loss of calcium‐activated BK potassium currents (Sausbier *et al*., 2004). However, in β_4_^−/−^ mice, AHP was not affected. Even so, prolonged inter‐spike intervals and reduced cortical output may contribute to the motor impairment of *lethargic* as well as of BK^−/−^ mice.

Overall, this study reveals both common and specific functions of β_4_ and Ca_v_2.1 in cerebellar networks. Considering the role of β_4_ as an auxiliary calcium channel subunit, the common deficiencies involving PF–PC PSCs and PC development suggest a role of the β_4_ isoform in modulating PSCs, pacemaker activity and dendritic maturation. In contrast, the absence of a phenotype in CF–PC synapse function and in PF–PC release probability indicates that for neurotransmitter release and synaptic competition β subunit functions may be redundant. Furthermore, the absence of β_4_‐specific defects of PC pacemaker firing regularity confirms an earlier study showing the redundancy of β_4_ at the PC soma (Burgess *et al*., 1999). Conversely, β_4_‐specific defects of PC pacemaker frequency suggest an essential function of β_4_ with a hitherto unidentified calcium channel isoform other than Ca_v_2.1. Finally, considering the role of β_4b_ as a regulator of gene transcription (Etemad *et al*., 2014), it is possible that loss of β_4_ may affect cerebellar development through calcium channel‐independent pathways. Taken together, the similarities and differences between β_4_^−/−^ and Ca_v_2.1 mutants suggests that Ca_v_2.1‐related and unrelated dysfunctions may coexist in the *lethargic* cerebellum.

### How does the loss of β_4_ affect the network activity in the cerebellar cortex of lethargic mice?

The observed reduction of the evoked PF input in cerebellar PCs is one of the most striking functional consequences of the loss of β_4_. In *lethargic* mice the cerebellar granular layer was thinner. This may imply a reduced number of granule cells and consequently fewer PFs. More importantly, the distal dendritic arbor of PCs in *lethargic* mice had a reduced height and width and with slightly sparser branching (Fig. 3; Fig. S1). According to our experimental paradigm, electrical stimulation of a given PF bundle would recruit a smaller dendritic area of *lethargic* PCs than in controls. This contributes to limiting the evoked PF–PC PSCs in *lethargic* mice. However, given the unchanged synaptic density (Fig. 3) and only mildly reduced distal dendritic density (Fig. S1) the morphological changes cannot fully account for the large reduction in the PF–PC PSC recorded in *lethargic* mice.

This explanation for the reduced PF volley is also challenged by previous research suggesting that other presynaptic and postsynaptic factors might contribute to aberrant neurotransmission upon loss of Ca_v_2.1 or β_4_ (Caddick *et al*., 1999; Lin *et al*., 1999; Wittemann *et al*., 2000; Kodama *et al*., 2006; Xie *et al*., 2007). However, in contrast to defects of neurotransmission found in Ca_v_2.1‐related pathologies (Pietrobon, 2002, 2005) and to what was observed upon loss of β_4_ in other neuronal systems (Lin *et al*., 1999; Wittemann *et al*., 2000; Xie *et al*., 2007), we found unaltered PF–PC release probability, suggesting that the loss of β_4_ in cerebellum did not severely affect presynaptic neurotransmitter release. This along with unaltered spine density of PF–PC synapses suggests a normal presynaptic physiology in the cerebellum of *lethargic* mice. The decreased PF–PC PSC could therefore be caused by specific impairment of postsynaptic function (Kodama *et al*., 2006) or directly related to the reduced distal branching of PC dendrites. Given the abundance of Ca_v_2.1 channels in PC dendrites and their function in the amplification of postsynaptic currents (De Schutter & Bower, 1994), a potential reduction of dendritic Ca_v_2.1 is expected to alter processing of the PF input. As postsynaptic enrichment of Ca_v_2.1 channels is an age‐dependent phenomenon and follows a somato‐dendritic gradient (Indriati *et al*., 2013), a loss of postsynaptic Ca_v_2.1 would well agree with the observed reduction of distal dendritic tree (Fig. S1), the decreased PF–PC input and the late onset of the *lethargic* phenotype. Specifically, dendritic spikes (Llinas *et al*., 1968; Llinas & Sugimori, 1980) may be affected by the postsynaptic loss of Ca_v_2.1 (Tank *et al*., 1988) in *lethargic* mice, which in turn would impair the processing and integration of PF and CF inputs (Rancz & Hausser, 2006; Davie *et al*., 2008), and long‐ and short‐term synaptic plasticity (Kreitzer & Regehr, 2001; Golding *et al*., 2002; Holthoff *et al*., 2004; Rancz & Hausser, 2006).

A reduced PC firing rate in adult *lethargic* mice was another striking effect of the loss of the β_4_ subunit in cerebellar cortex. Because PF and CF input have antagonistic effects on PC excitability and pacemaker rates (Shibuki & Kimura, 1997; Smith & Otis, 2003; Cerminara & Rawson, 2004), the skewed proportion between PFs and CFs observed here in *lethargic* mice might dictate reduced rates of pacemaker firing in PCs. However, PC firing frequency was not affected by the acute block of tonic neurotransmission, thus questioning a causal link between reduced PF input and reduced PC pacemaker activity in the *lethargic* cerebellum.

### How do defects in the cerebellar network contribute to the motor impairment in lethargic mice?

Our slice electrophysiology analysis of the cerebellar network in *lethargic* mice revealed deficiencies at the input and output side of PCs, both of which could contribute to ataxia. Motor learning relies on the plasticity of PCs and their role in integrating the inputs from PFs and CFs (Ito *et al*., 1982; Hansel & Linden, 2000; Ito, 2001). In *lethargic* mice evoked PF PSCs are specifically reduced, which could impair motor learning (Galliano *et al*., 2013). In the presynaptic compartment this could be caused by a paucity of granule cells from which the PFs originate, and in the postsynaptic compartment by smaller medial and distal PC dendrites onto which PFs project. Yet, the CF input and proximal PC dendrites are unaffected by the loss of β_4_. The resulting imbalance between PF and CF input onto PCs by itself could account for impaired plasticity, learning and coordination (Ito *et al*., 1982; Hansel & Linden, 2000; Ito, 2001; Galliano *et al*., 2013). Similarly, defective dendritic processing of both PF and CF inputs could lead to impaired network plasticity (Kreitzer & Regehr, 2001; Golding *et al*., 2002; Holthoff *et al*., 2004; Rancz & Hausser, 2006). Ultimately, cerebellar dysfunctions must be reflected in altered PC tonic output. As PCs represent the sole cerebellar cortical output, the observed slowed PC pacemaker firing will result in inefficient inhibition of the deep cerebellar nuclei (Gauck & Jaeger, 2000). Similarly reduced tonic output has previously been described in other mouse models with impaired motor coordination (Sausbier *et al*., 2004). Thus, reduced tonic firing of cerebellar PCs could be a major cause of the motor impairment generated by the loss of the calcium channel β_4_ subunit in *lethargic* mice.

## Conflict of interest

The authors declare no competing financial interests.

## Supporting information

Fig. S1. Substantially reduced size and modestly reduced density of the distal dendritic arbor in *lethargic* Purkinje neurons.
