# Supplementary material for: Loss of the calcium channel β4 subunit impairs parallel fibre volley and Purkinje cell firing in cerebellum of adult ataxic mice
Source: Eur J Neurosci. 2016 Apr 18;43(11):1486–98. doi: 10.1111/ejn.13241 (PMC4949674; doi:10.1111/ejn.13241)
Supplement: Supplementary file 1 — Fig. S1. Substantially reduced size and modestly reduced density of the distal dendritic arbor in lethargic Purkinje neurons. [file EJN-43-1486-s001.pdf]

Supporting information

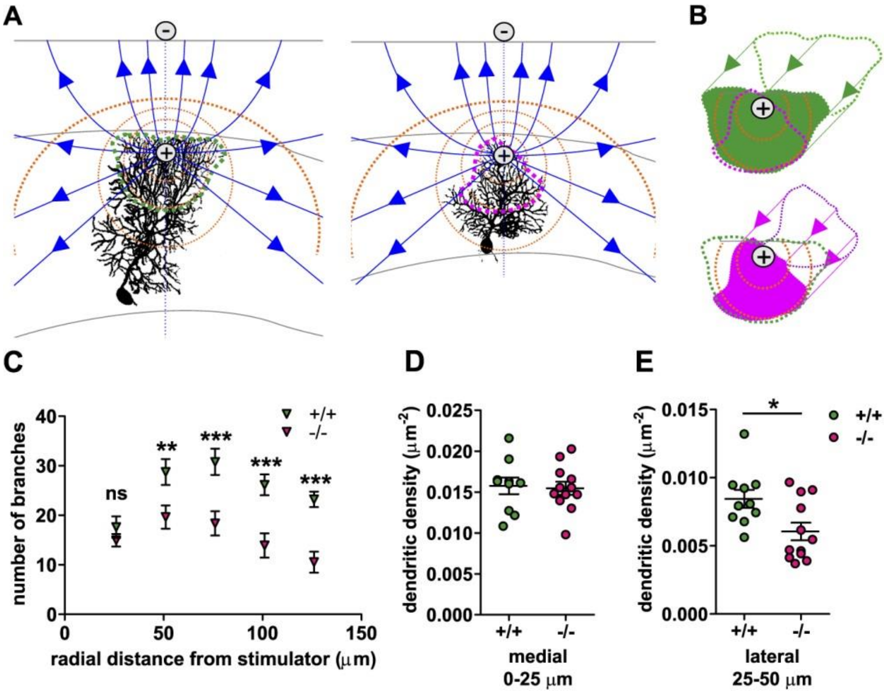

Supplementary Fig. 1: Substantially reduced size and modestly reduced density of the distal dendritic arbor in *lethargic* Purkinje neurons. A. Schematic models of unipolar stimulation (Galliano et al. 2013) of parallel fibers innervating the apical dendrites of *wildtype* (left) and *lethargic* (right) mice. Blue lines represent the electric field and orange circles the equipotential lines relative to the contour of dendritic arbors; the intersection with a given equipotential line is outlined in green (+/+) or pink (-/-). **B.** Contours of dendritic arbors expanded from **A** in which filled areas represent the putative projection areas for bundles of Parallel Fiber afferents (arrows). Note that stimuli of a given strength will activate synapses over a larger projection area in *wildtype* neurons (green) than in *lethargic* neurons (pink). To test this model we counted apical dendrites within concentric semicircles, using the pial surface as base and the position "+" as center-point of our Scholl-analysis circles. Thus, dendritic arbors were aligned at their outmost apical branches rather than at the soma (as in Fig. 3.C). **C.** Sholl analysis shows a significant increase in the number of distal branches in +/+ neurons (n = 13) compared to -/- neurons (n = 10). Note that there is no difference in the

number or density of dendrites at the center of Purkinje cell arbors (smallest circle, corresponding to weak stimulation) whereas in distal dendrites (circles > 25µm) the dendritic arbor is sparser and thinner in -/- neurons. **D**. Density plot (number of dendrites / circle area) shows that the dendrite density is equal in the medial portion of the *lethargic* PC dendritic arbor compared to *wildtype* (P = 0.82). **E**. The density plot shows a modest reduction in the lateral dendritic density in the *lethargic* cerebellum (P = 0.018). Based on previous measurements (Benedetti et al., 2011) we estimate that in current experiments the maximal intensity of stimulation may recruit a bundle of Parallel fibers across a semicircular section of approximately 100 µm radius from the pial surface. According to our model, at each given radial distance (i.e. equipotential lines) larger than 25 µm from the cathode, the electric field will encounter fewer dendrites in -/- than in +/+ neurons. This may contribute to the larger PF-PC PSC observed in +/+ mice compared to that observed in -/- mice (**Fig.2**). In **B** statistical comparison was performed using the two-way Anova test, followed by Bonferroni Posttest. In **C** and in **D** populations were compared with unpaired T test. *: P < 0.05, **: P < 0.01, **: P < 0.0001.
